# Nanoplasmonic Avidity-Based Detection and Quantification of IgG Aggregates

**DOI:** 10.1021/acs.analchem.2c03446

**Published:** 2022-11-01

**Authors:** Thuy Tran, Erik Martinsson, Sergio Vargas, Ingemar Lundström, Carl-Fredrik Mandenius, Daniel Aili

**Affiliations:** † Laboratory of Molecular Materials, Division of Biophysics and Bioengineering, Department of Physics, Chemistry and Biology, Linköping University, Linköping 581 83, Sweden; ‡ ArgusEye AB, Spannmålsgatan 55, Linköping 583 36, Sweden; § Wolfram MathCore AB, Teknikringen 1E, Linköping 583 30, Sweden; ∥ Sensor and Actuator Systems, Department of Physics, Chemistry and Biology, Linköping University, Linköping 581 83, Sweden; ⊥ Biotechnology, Division of Biophysics and Bioengineering, Department of Physics, Chemistry and Biology, Linköping University, Linköping 581 83, Sweden

## Abstract

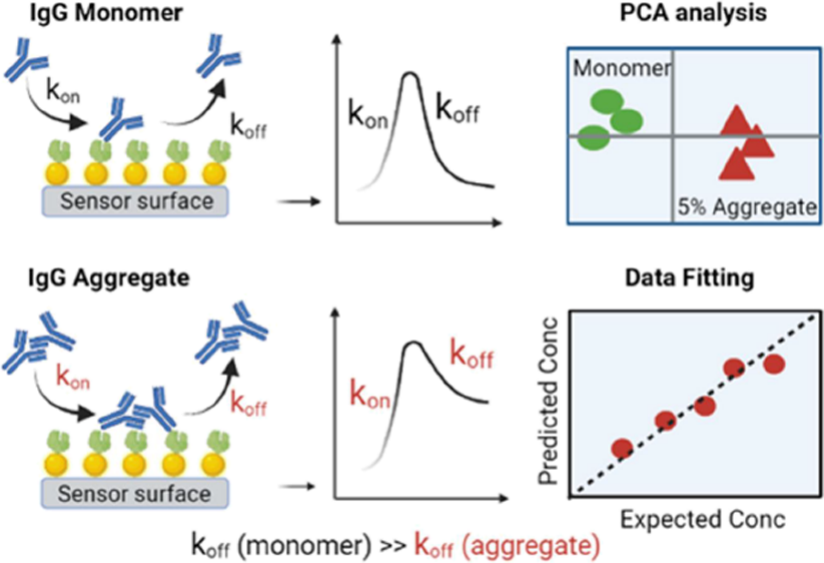

Production of therapeutic monoclonal antibodies (mAbs) is a complex process that requires extensive analytical and bioanalytical characterization to ensure high and consistent product quality. Aggregation of mAbs is common and very problematic and can result in products with altered pharmacodynamics and pharmacokinetics and potentially increased immunogenicity. Rapid detection of aggregates, however, remains very challenging using existing analytical techniques. Here, we show a real-time and label-free fiber optical nanoplasmonic biosensor system for specific detection and quantification of immunoglobulin G (IgG) aggregates exploiting Protein A-mediated avidity effects. Compared to monomers, IgG aggregates were found to have substantially higher apparent affinity when binding to Protein A-functionalized sensor chips in a specific pH range (pH 3.8–4.0). Under these conditions, aggregates and monomers showed significantly different binding and dissociation kinetics. Reliable and rapid aggregate quantification was demonstrated with a limit of detection (LOD) and limit of quantification (LOQ) of about 9 and 30 μg/mL, respectively. Using neural network-based curve fitting, it was further possible to simultaneously quantify monomers and aggregates for aggregate concentrations lower than 30 μg/mL. Our work demonstrates a unique avidity-based biosensor approach for fast aggregate analysis that can be used for rapid at-line quality control, including lot/batch release testing. This technology can also likely be further optimized for real-time in-line monitoring of product titers and quality, facilitating process intensification and automation.

## 1 Introduction

Therapeutic monoclonal antibodies (mAbs) have been the predominant segment of approved therapeutic proteins over the past several years and still have significant potential for growth.^1,2^ In 2021, six out of 14 protein-based drugs approved by the US Food and Drug Administration (FDA) were mAbs.^3^ These new drugs show high efficiency and safety in disease treatments, but treatment costs tend to be very high. The highly complex and time-consuming production processes of biopharmaceuticals are factors contributing to the high costs.^4^ Aggregation of mAbs during the manufacturing process has been shown to reduce their therapeutic efficacy and enhance immunogenicity, causing several adverse side effects, and is thus a critical quality attribute in antibody bioproduction.^5−8^ The aggregates can be of various sizes, including small soluble oligomers (dimers, trimers, tetramers, etc.) as well as larger visible or subvisible nonsoluble aggregates that can be removed by 0.22 μm filtration or mild centrifugation.^9^

During mAb manufacturing, depending on the mAb characteristics, bioprocessing strategies, and external stressors, aggregate levels can vary from about 0.5 to 60%.^10,11^ Aggregate levels of 26 commercial therapeutic mAbs and 4 related products analyzed using size exclusion chromatography (SEC) were reported to vary from 0.1 to 13% in the final formulation, and most of these samples had aggregate levels higher than 0.3%.^12^ There are no general regulatory limits for soluble aggregates levels in protein-based pharmaceutical products, and the acceptable maximum concentrations must be set on a case-by-case basis to maintain safety and efficacy of the product.^13,14^ Avoiding aggregate formation, however, remains a major challenge, and improved process understanding, efficient aggregate monitoring, and removal strategies are essential aggregate management strategies in order to ensure the quality of the product.^9^

While several methods have been reported for qualitative and quantitative analysis of soluble protein aggregates (up to 100 nm), such as dynamic light scattering (DLS), nanoparticle tracking analysis (NTA), differential scanning fluorimetry (DSF), and transmission electron microscopy (TEM), SEC has been the gold standard technique for mAb aggregate characterization.^15,16^ Modern analytical SEC instruments, combined with multiple detectors such as multi-angle light scattering (MALS) or mass spectrometry (MS), can provide extensive information on product and aggregate concentrations and molar masses without the need for column calibration.^17^ Sedimentation velocity analytical ultracentrifugation (SV-AUC) is also widely used for the characterization of antibody aggregates, including small soluble oligomers, because of its wide dynamic range and high reliability.^18,19^ However, both SEC and SV-AUC are time-consuming and require complicated instrumentation and substantial expertise to operate. In addition, neither of these techniques are suitable as process analytical technology (PAT) tools^20,21^ as they do not allow for rapid at-line, on-line, or in-line measurements of aggregates during mAb production or purification. Despite significant efforts to develop new techniques with a potential for rapid or real-time process monitoring, including Raman spectroscopy,^22,23^ a real-time PAT tool for the analysis of protein aggregates has not yet been demonstrated.

**Figure 1:**
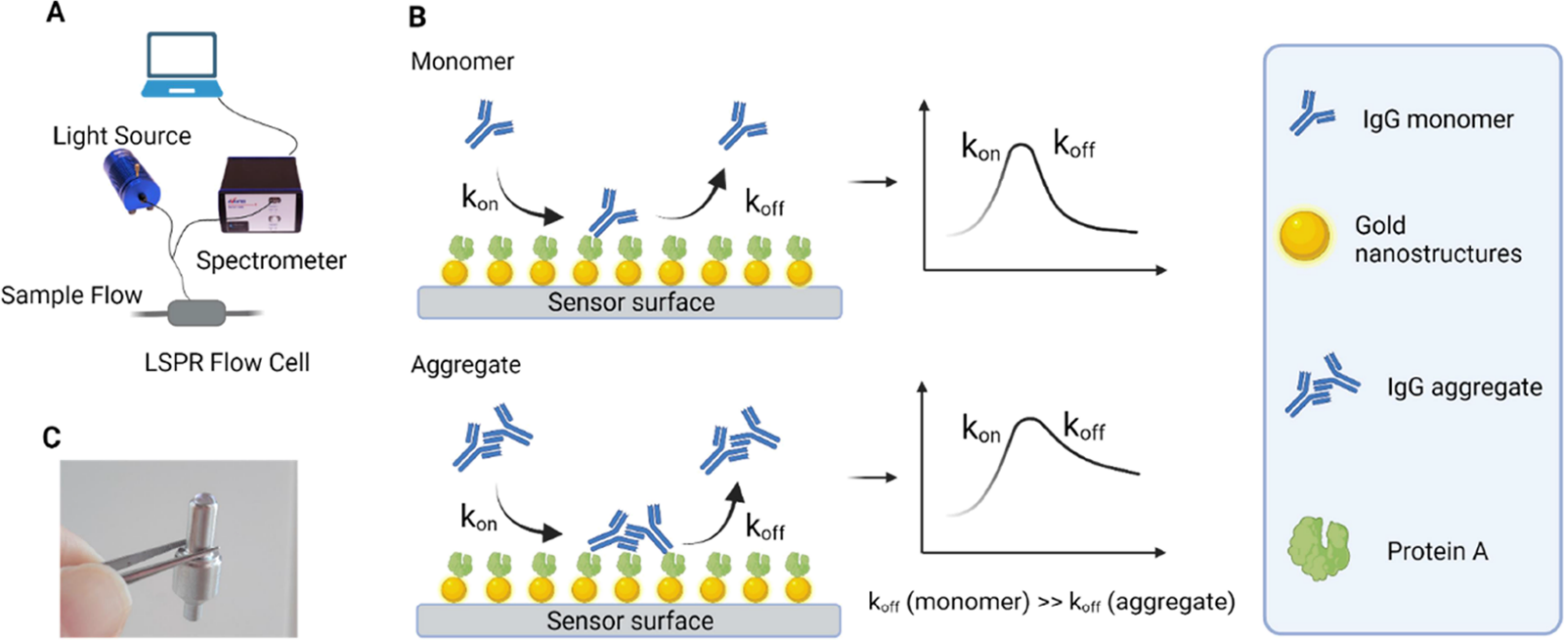
(A) Illustration of the LSPR biosensor setup. (B) Illustration of the binding of monomers and aggregates to the sensor surface containing gold nanostructures functionalized with Protein A. IgG monomers and aggregates have different molecular weights and sizes. Larger IgG aggregates can bind to multiple Protein A immobilized on the sensor chip. Multivalent interactions result in a slower dissociation rate (*k*_off_) for aggregates compared to monomers. (C) Photograph of a functionalized sensor chip.

Here, we show a unique bioanalytical strategy for detection and quantification of mAb aggregates based on specific analyte recognition using a novel localized surface plasmon resonance (LSPR)-based biosensor. This sensor technology (Figure 1A) has recently been demonstrated to enable rapid, reproducible, and reliable quantification of IgG titers in both upstream and downstream process steps.^24^ LSPR is the result of collective electron oscillations in noble metal nanoparticles, such as gold nanoparticles (AuNPs), upon irradiation with visible light, resulting in a pronounced extinction band. The position of this so called LSPR band is highly dependent on the refractive index in the vicinity of the nanoparticle surface. Binding of mAbs to ligands (e.g., Protein A) immobilized on the nanoparticle surface results in a concentration-dependent shift of the LSPR band that can be measured spectroscopically. This optical phenomenon is related to surface plasmon resonance (SPR), which is used in several different high-performing benchtop biosensor instruments for biomolecular interaction analysis. Both LSPR and SPR are label-free techniques that can monitor analyte binding to ligands immobilized on a sensor surface (sensor chip). However, whereas SPR is highly sensitive to fluctuations in temperature and sample matrix composition, LSPR-based sensors are more surface-sensitive and can operate under ambient conditions.^25,26^ When combined with an appropriate and robust surface chemistry on the sensor chip, specific analyte detection in very complex samples is possible.^27^ The sensor signal depends on the molecular weight and concentration of the analytes and the analyte–ligand affinity. When the analyte interacts with multiple ligands immobilized on the sensor chip, the strength of the accumulated interactions is seen as an enhanced apparent affinity. We hypothesize that this so-called avidity effect is likely to occur for IgG aggregates (dimers, trimer, and higher oligomers) because the oligomers can interact with multiple IgG-binding ligands (Protein A) on the sensor chip. As a result, the association (*k*_on_) and dissociation (*k*_off_) rate constants for monomeric IgG and IgG aggregates will differ, as illustrated in Figure 1B.

The sensor system used here comprises a flow cell that can be connected either directly to a chromatography system or to a separate liquid handling system with a pump. The sensor chip is inserted into the flow cell and connected to an optical unit for sensor readout using fiber optics. The low foot-print optical unit contains a white light source for exciting the LSPR and a detector for monitoring changes in the LSPR band upon analyte binding. Here, we functionalized the LSPR sensor chips with Protein A, which is a common IgG binding ligand, and investigated the binding and dissociation kinetics of samples containing IgG monomers and aggregates at different concentrations and ratios.

Indeed, IgG aggregates were found to bind with higher apparent affinity compared to monomers, which enabled rapid detection and quantification of aggregates. By careful analysis of both the total response and the kinetic profile, we could detect and quantify aggregates with a limit of detection (LOD) and limit of quantification (LOQ) of 9 and 30 μg/mL, respectively, within a few minutes. In addition, simultaneous measurements of monomer and aggregate concentrations could be achieved. To our knowledge, this is the first demonstration of a biosensor exploiting avidity effects combined with advanced data analysis for quantification of IgG variants. This novel biosensor technology offers rapid analysis of mAb quality for quality control, batch and lot release testing, or bioprocess monitoring that can significantly reduce hold times and increase process efficiency, resulting in more cost-effective production of biopharmaceuticals. The robust and flexible sensor technology can further improve process understanding and facilitate development of strategies for on-line or in-line detection of aggregates in both upstream and downstream process steps to enable process automation and intensification.

## 2 Experimental Section

### 2.1 Reagents and Materials

N-Ethyl-N′-(3-dimethylaminopropyl)carbodiimide (EDC), N-hydroxysuccinimide (NHS), 4-morpholineethanesulfonic acid (MES), ethanolamine, sodium citrate dihydrate, citric acid, and glycine were obtained from Sigma-Aldrich (St. Louis, MO, USA). Protein A and phosphate buffered saline (PBS) tablets were supplied by Medicago AB (Upsala, Sweden). LSPR sensor chips were provided by ArgusEye AB (Linköping, Sweden). Human IgG1 and mouse IgG2a were produced by BioInvent International AB (Lund, Sweden). Mouse IgG2a was purified using a Protein A capture step and collected as a mixture of both monomers and aggregates. The aggregate content was 5.7% as determined by SEC. The produced IgG1 batch contained a natural distribution of both monomers and aggregates caused by normal processing conditions. Aggregates and monomers were separated and purified using size exclusion chromatography high performance liquid chromatography (SEC-HPLC), resulting in a concentration and purity of 4.5 mg/mL, 99% purity (monomer fraction) and 1.5 mg/mL, 97% purity (aggregate fraction), respectively, which was further verified using SEC-MALS as described below.

### 2.2 Characterization of IgG Aggregates Using SEC

Monomer and aggregate contents of samples were verified using a SEC setup with Multi-Angle Static Light Scattering, Refractive Index, and UV detection (SEC-MALS-RI-UV). The system includes an Agilent HPLC 1100 system with a UV–vis diode array detector coupled with DynaPro Nanostar dynamic light scattering, miniDAWN TREOS multi-angle light scattering, and Optilab T-rEX refractive index detectors (Wyatt Technology, Santa Barbara, CA). Refractive index change was measured differentially with a GaAs laser at a wavelength of 690 nm, and UV absorbance was measured with the diode array detector at 280 nm. A Superdex 200 Increase 10/300 GL column was used for the separation of monomers and aggregates. The flowrate was set at 1 mL/min, and 100 μL of samples was injected for all measurements. The column was kept at room temperature. The Agilent software was used to control the HPLC, and the Wyatt Astra software was used for data collection and analysis. Peak alignment and band broadening correction between the UV, MALS, and RI detectors were performed using the Astra software algorithms. Percentages of aggregate present in the samples were validated based on UV signals.

### 2.3 Ligand Immobilization

Carbodiimide (EDC/NHS) coupling chemistry was used to immobilize Protein A on the sensor chips. A (v/v 1:1) mixture of 20 μL of 0.4 M EDC and 0.1 M NHS was added to the sensor chips and incubated for 45 min. After rinsing with Milli-Q water (18.2 MΩ cm^–1^), 20 μL of 0.5 mg/mL Protein A solution was added and incubated for 2 h. Deactivation of unreacted active esters was performed using 20 μL of 1 M ethanolamine (pH 8.5) for 30 min. The sensor chips were rinsed and stored in PBS buffer (140 mM sodium chloride, 2.7 mM potassium chloride, and 10 mM phosphate) pH 7.4, before being inserted into the LSPR system.

### 2.4 LSPR Measurements

Sensorgrams were collected using a fiber optical sensor system provided by ArgusEye AB (Linköping, Sweden). The LSPR system comprises a white light source, optical detection unit, and a flow cell. Sensor chips functionalized with Protein A were docked into the flow cell and equilibrated with PBS buffer using an HPLC pump for about 3 min before sample injection. Samples were injected into the flow cell through an injection valve, and sensor responses were continuously recorded using the ArgusEye software. A 1-min pulse of regeneration buffer (10 mM Glycine-HCl pH 2.5) was used to regenerate the sensor chips between sample injections. All experiments were performed at room temperature under ambient conditions. Running buffers were PBS (pH 7.4) and 10 mM citrate supplemented with150 mM NaCl and adjusted to different pH values (pH: 4.0, 3.8, and 3.5). For kinetic measurements, samples were prepared in the same running buffer, and for detection and quantification, the samples were diluted in PBS.

### 2.5 Prediction Models for Aggregate and Monomer Concentrations

To generate a prediction model for identifying aggregate and monomer concentrations, a dimensional reduction approach on the sensor response curve was used. Briefly, sample responses were fitted to an exponential model and the corresponding fit parameters were used as input data in the prediction of aggregate and monomer concentrations. This approach was applied for association and dissociation curves using two exponential equations, *a* + *be*^–*ct*^ and *d* + *fe*^–*gt*^, respectively, producing three fit parameters each. These parameters were then used as input for training a neural network to predict the corresponding aggregate and monomer concentrations. In the training, the concentrations were passed through a logarithm transformation for standardization of output data. The neural network had seven layers; four linear alternating with three scaled exponential linear units (SELU). Validation using 14 data sets was caried out using Mathematica,^28^ using functions such as NonlinearModelFit and NetTrain. More details on the neural network training are provided in the Supporting Information.

## 3 Results and Discussion

### 3.1 Characterization of Monomer and Aggregate Samples Using SEC

To develop and evaluate the sensor technology and produce training data sets for the neural network, IgG samples with a well-known concentration and distribution of monomers and aggregates were produced. The samples used here were obtained from the same production batch, comprising a natural distribution of monomers and aggregates. No additional stressors to trigger aggregate formation were applied. Aggregates and monomers were purified using SEC-HPLC into a monomer and aggregate fraction. Protein aggregates have been reported to be sensitive to storage conditions^29^ and can, in some cases, dissociate into smaller aggregates and even monomers when reducing the concentration by diluting the samples.^30^ The monomer and aggregate content as well as size distribution of aggregates were therefore analyzed using a SEC-MALS detector to obtain accurate molar mass profiles and purities of the samples prior use. SEC chromatograms of samples (0.45 mg/mL total IgG concentration) from the monomer and aggregate fractions and a mixture prepared to contain 20% aggregates and 80% monomers are shown in Figure S1A,B (Supporting Information). The samples from the aggregate fraction were found to contain mainly IgG dimers and a negligible level of trimers and oligomers. The purity of the monomer and aggregate samples were found to be 98.9 and 97.8%, respectively. Formation of larger subvisible aggregates was not observed. New samples with desired amounts of monomers and aggregates for use in further experiments were prepared based on the purities obtained from the SEC analysis.

### 3.2 Binding of IgG1 Monomers and Aggregates to Protein A Sensor Chips at Different pH

**Figure 2:**
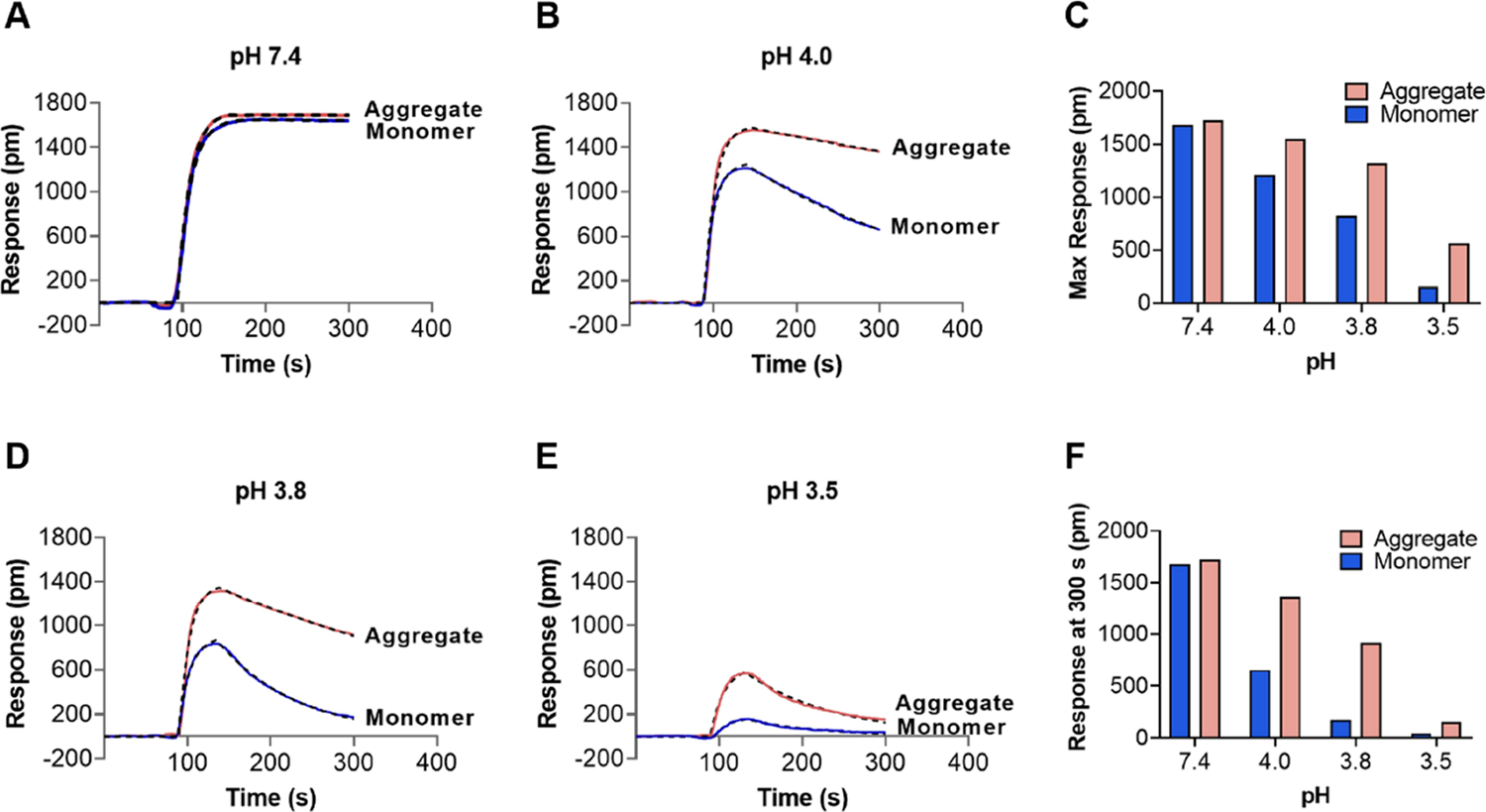
Effect of pH on binding and dissociation of IgG monomers and aggregates. (A) and (B) LSPR sensograms at pH 7.4 and pH 4.0, respectively. (C) Maximum binding responses at different pH values. (D) and (E) LSPR sensograms at pH 3.8 and pH 3.5, respectively. (F) Responses at 300 s at different pH values. Dashed black curves are fitting sensorgrams using a 1:1 Langmuir binding model. In all experiments, 1 mL of samples with total concentrations of 0.45 mg/mL was injected at a flow rate of 1 mL/min and one injection (*n* = 1) was performed.

Protein A is a well-known immunoglobulin binding protein and has a very high affinity for binding of IgGs, such as human IgG1, IgG2, and IgG4, typically with an equilibrium constant *K*_D_ ∼ 2 × 10^–9^ M.^31^ The binding is optimal at pH 7.5–8 and is disrupted at pH ≤ 3.5.^32,33^ To investigate whether IgG monomers and aggregates show different binding and dissociation kinetics to the Protein A sensor chips, sensorgrams at physiological as well as mildly acidic pH values were recorded. The monomer and aggregate samples used had identical concentrations of IgG, as determined by UV spectroscopy. LSPR sensorgrams were recorded for samples containing only IgG monomers (<1% aggregates) or only IgG aggregates at pH 7.4, 4.0, 3.8, and 3.5 (Figure 2A,B,D,E, respectively). Not surprisingly, the interaction between Protein A and both IgG monomers and aggregates was clearly pH-dependent. It was noteworthy that while the binding profiles of monomers and aggregates at pH 7.4 (Figure 2A) were rather similar, they were significantly different at the three lower pH values (4.0, 3.8, and 3.5) (Figure 2B,D,E). The maximum response decreased, and the dissociation rate increased for both monomers and aggregates at pH ≤ 4 compared to that at pH 7.4 (Figure 2C). In addition, the response at the end of the dissociation phase (at 300 s) also decreased when lowering the pH (Figure 2F).

Interestingly, samples with aggregates were found to have higher maximum binding response (Figure 2C) and higher response at the end of the dissociation phase (Figure 2F) at acidic pH values compared to monomers. These differences were most pronounced at pH 4.0 and 3.8 and clearly show that IgG aggregates dissociate slower compared to monomers, indicating a stronger binding of IgG aggregates to the Protein A sensor chips because of avidity effects caused by IgG aggregates binding to multiple Protein A molecules. The multivalent interactions might occur as a result of binding to several Protein A molecules on a single nanostructure or to Protein A molecules immobilized on two or more adjacent nanostructures. Considering the size of Protein A (∼2.5 nm) and the size of the gold nanostructures on the sensor chip (∼50 nm), each nanostructure could in theory carry more than 800 Protein A molecules if closely packed on the surface. However, even with just 10% Protein A surface coverage, there will be multiple IgG-binding ligands available on each discrete nanostructure. In addition, Protein A has five homologous Fc-binding domains.^31,34^ These binding domains have also been demonstrated to interact with the Fab region of human IgG.^35,36^ Therefore, with several potential IgG binding domains positioned closely on the surface of the sensor chip, there is a high probability for multivalent interactions where IgG aggregates can interact with multiple immobilized Protein A molecules, resulting in pronounced avidity effects. For larger aggregates, we expect that the avidity effects would be even more pronounced because of the larger number of possible interactions between the immobilized Protein A and the aggregate. Oxidation of methionine residues in the Protein A binding Fc region of IgG1 (Met257 and Met433) can, however, also influence Protein A binding affinity.^37^ In the current study, all samples were subject to the same treatment and storage conditions. Thus, no differences in oxidation between monomer and aggregates samples were expected that could influence the interpretation of the results.

### 3.3 Data Fitting and Comparison of Binding Kinetics

We further evaluated the binding kinetics of the IgG monomer and dimeric aggregate by fitting the observed binding curves to a Langmuir 1:1 binding model^38^ (Figure 2). The model and fitting procedure is described in detail in the Supporting Information. The fitting shows very good correlation between the model and the responses (*R*^2^ ≥ 0.98 for curve fittings). An example of observed and fitted sensorgrams, curve fittings with equations and *R*^2^ values, and the residual plot for IgG binding at pH 3.8 is shown in Figure S2 (Supporting Information). The larger residuals obtained for the first part of the binding curve could be due to slight differences between the refractive indexes of the sample and the buffer, which can be seen as a small negative response at the beginning of the binding phase. The obtained association (*k*_on_) and dissociation (*k*_off_) rate constants and (apparent) affinity (*K*_D_) are presented in Table 1.

**Table 1:** LSPR Binding Kinetics and Affinities for IgG Monomers and Aggregates Binding to Protein A Sensor Chips at Different pH Values Obtained Using a Langmuir 1:1 Binding Model

	monomers (M)			aggregates (A)			monomers versus aggregates		
	*k*_on_ (M^–1^ s^–1^)	*k*_off_ (s^–1^)	*K*_D_ (M)	*k*_on_ (M^–1^ s^–1^)	*k*_off_ (s^–1^)	*K*_D_ (M)	*k*_on_ M/A	*k*_off_ M/A	*K*_D_ M/A
pH 7.4	2.2 × 10^4^	2.9 × 10^–5^	1.3 × 10^–9^	2.5 × 10^4^	2.9 × 10^–5^	1.2 × 10^–9^	0.9	1.0	1.2
pH 4.0	3.1 × 10^4^	4.0 × 10^–3^	1.3 × 10^–7^	3.6 × 10^4^	9.2 × 10^–4^	2.6 × 10^–8^	0.9	4.3	5.0
pH 3.8	3.0 × 10^4^	1.0 × 10^–2^	3.3 × 10^–7^	3.6 × 10^4^	2.4 × 10^–3^	6.5 × 10^–8^	0.8	4.2	5.1
pH 3.5	1.7 × 10^4^	1.1 × 10^–2^	6.6 × 10^–7^	2.8 × 10^4^	9.9 × 10^–3^	3.5 × 10^–7^	0.6	1.1	1.9

The association rates (*k*_on_) of both monomer and aggregate samples at pH 7.4, 4.0, 3.8, and 3.5 were of the same order of magnitude, ranging from 1.7 × 10^4^ to 3.6 × 10^4^ M^–1^ s^–1^ and are in good agreement with *k*_on_ values at pH 7.4 demonstrated in other reports.^39,40^ Very slow *k*_off_ (2.9 × 10^–5^ s^–1^) and high (apparent) affinity (*K*_D_ = 1.3 × 10^–9^ M) of IgG at pH 7.4 also agreed well with the well-known strong binding between Protein A and human IgG (*K*_D_ ∼ 2 × 10^–9^ M).^31^ At pH 7.4, aggregates and monomers had similar binding kinetics, and their binding responses typically reached the maximum binding capacity of the sensor chip, indicating similar surface concentrations of IgG molecules on the sensor surface in both cases. This observation most likely suggested that the aggregates, which were mostly dimers, interacted with or at least blocked two ligand sites on the sensor surface. It is also possible that some of the dimers blocked only one ligand site but orienting the dimer partly outside the LSPR sensing depth, therefore generating a similar response as a monomer. The sensing depth of the LSPR nanostructures used here is about 15–20 nm.^41,42^ A single IgG molecule (∼12 nm) bound to a protein A ligand (∼2.5 nm) consequently occupies almost the entire sensing depth.

When comparing dissociation rates (*k*_off_) of monomers and aggregates, only minor differences were seen for the two species at pH 7.4 and 3.5. At pH of 3.5, most IgG molecules had dissociated from the sensor surface after 300 s with similar dissociation rates for both monomers and aggregates. In contrast, at pH 3.8 and 4.0, the *k*_off_ values for aggregates were approximately four times lower than those for monomers. Consequently, the overall (apparent) affinity (*K*_D_) for binding of IgG aggregates to the ligands on the sensor surface appeared to be enhanced about five times for aggregates, indicating pronounced avidity due to multivalent binding. Thus, it was apparent that IgG aggregates were bound more tightly to the sensor surface than the corresponding monomers. The greater apparent affinity can likely be explained by the accumulated binding strength by multiple binding sites involved in the interaction between IgG aggregates and the immobilized ligands. These findings are also consistent with other reports, demonstrating avidity effects upon binding of IgG oligomers to Fc receptors.^43,44^

### 3.4 Effects of Aggregate Levels on Ligand Binding Properties

**Figure 3:**
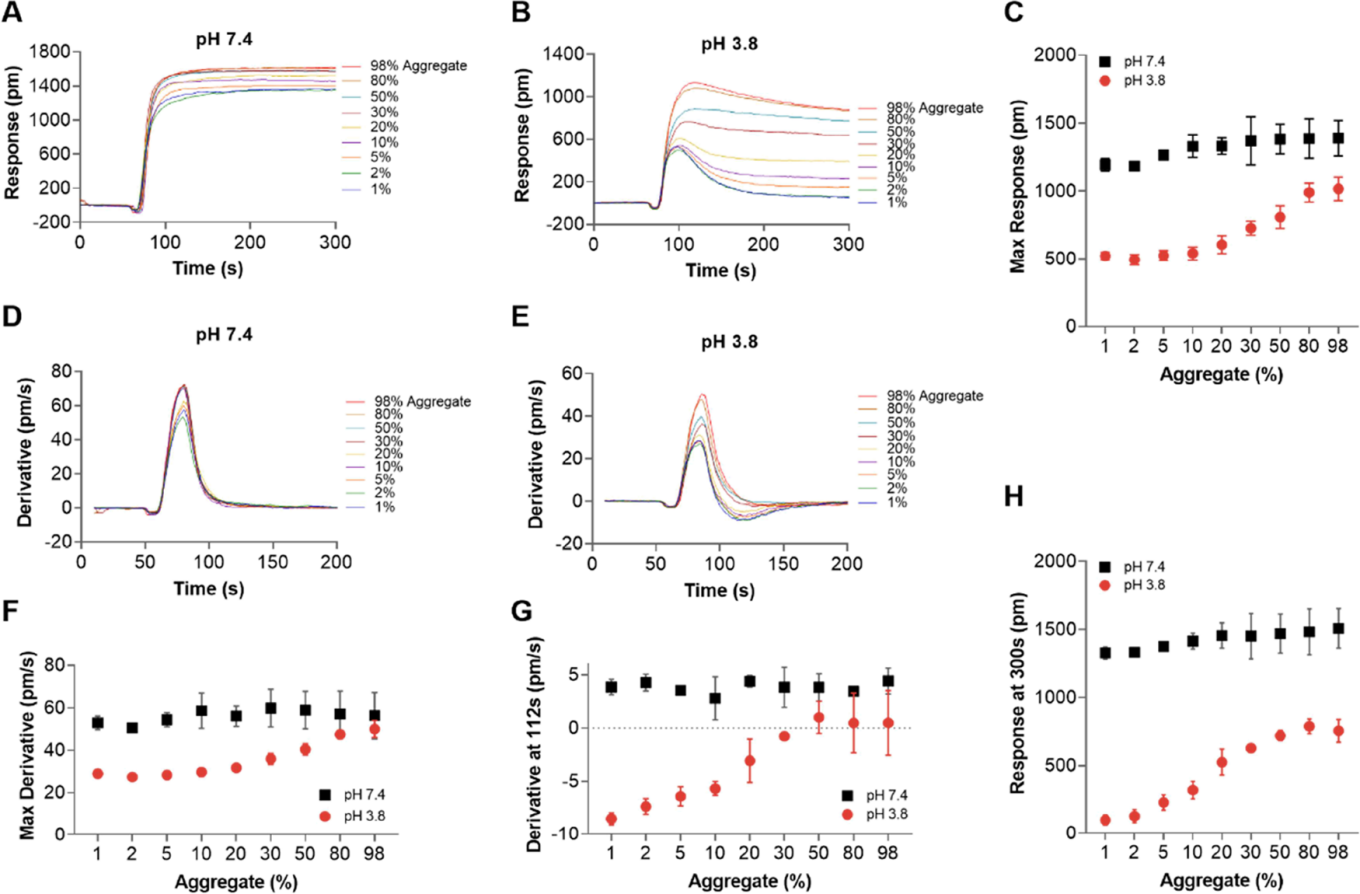
(A) and (B) Sensorgrams of nine different samples with various aggregate levels (1 to 98%) using PBS pH 7.4 and citrate 50 mM, 150 mM NaCl, pH 3.8 as running buffers. (C) Maximum response versus aggregate level. (D) and (E) Derivative curves of sensorgrams in (A) and (B), respectively. (F) Maximum derivative and (G) derivative at 112 s versus aggregate level. (H) Response at 300 s versus aggregate level at pH 7.4 (black square) and pH 3.8 (red circle), *n* = 2, error bars: standard deviation. In all experiments, 0.1 mL of samples with a total concentration of 0.45 mg/mL was injected at a flow rate of 1 mL/min.

To explore the effect of aggregate levels on the binding and dissociation curves, nine monomer samples spiked with different amounts of aggregates, ranging from 1 to 98%, were injected into the LSPR system using two different running buffers, PBS pH 7.4 and 10 mM citrate pH 3.8. As shown by SEC, even the monomer fraction contained some aggregates, although at a rather low concentration (1%). Samples prepared using only the monomer fraction are consequently referred to as 1% aggregate from here on. Binding/dissociation curves for the samples with different aggregate contents and their corresponding first derivatives are shown in Figure 3A,B,D,E, respectively.

By visual inspection of the data, the differences between samples with different aggregate contents were clearly more distinct when using a pH 3.8 running buffer compared to those at pH 7.4. At pH 7.4, samples only differed with respect to the maximum binding response and magnitude of their derivative, whereas at pH 3.8, samples with different compositions could also be identified from the large differences in the dissociation phase and corresponding derivatives. Maximum responses and the responses at 300 s (i.e., at the end of the dissociation phase) plotted against the aggregate levels are shown in Figure 3C,H, respectively. The presence of aggregates had a larger effect on both the maximum responses and the response at 300 s at pH 3.8 compared to that at pH 7.4. Noticeably, at pH 3.8, a clear difference between the sensor responses from samples with 5 and 1% aggregates was seen, even at the relatively low total IgG concentration used here (0.45 mg/mL) (Figure 3C,H). The increase in these responses due to higher amounts of aggregates could be explained by the greater apparent affinity (∼5 times) for aggregates (*K*_D_ = 6.5 × 10^–8^ M) compared to monomers (*K*_D_ = 3.3 × 10^–7^ M) when binding to the sensor chips, as discussed above (Table 1). Similarly, the presence of aggregates had very minor or no effect on the maximum derivatives (Figure 3F) and derivatives at the beginning of the dissociation phase (at 112 s) (Figure 3G) at pH 7.4. In contrast, at pH 3.8, derivatives at 112 s were greatly increased, from −8.5 for 1% aggregate to 0 for 98% aggregate, confirming the slower dissociation of aggregates compared to monomers.

We further investigated the sensor response when exposed to two different types of IgGs by comparing samples of human IgG1 with 5% of aggregates and mouse IgG2a containing 5.7% aggregates. The sensorgrams (Figure S3) showed similar characteristics for both samples with respect to both the maximum response and the dissociation rate, indicating that the avidity effect was not limited to human IgG1. The slightly higher binding response at 300 s for mouse IgG2a could potentially be due to the slightly higher aggregate content in this sample.

### 3.5 Aggregate Detection and Quantification

To further improve possibilities to detect aggregates, we used principal component analysis (PCA), focusing on the three samples with the lowest levels of aggregates, 1, 2, and 5% (Figure S4). The result showed that samples containing 1 and 2% aggregates clustered into the same group on the negative side of PC1, while all samples with 5% aggregates appeared in the positive area (score plot, Figure S4C). Binding responses from 190 to 300 s were higher for samples with 5% aggregates compared to samples with 1–2% aggregates (loading plot, Figure S4D). Derivatives from 110–122 s gave similar but smaller contributions to the group separation. The PCA analysis consequently allows for detection and discrimination of aggerate levels down to 5% at a total IgG concentration of 0.45 mg/mL, corresponding to an aggregate concentration of 23 μg/mL.

**Figure 4:**
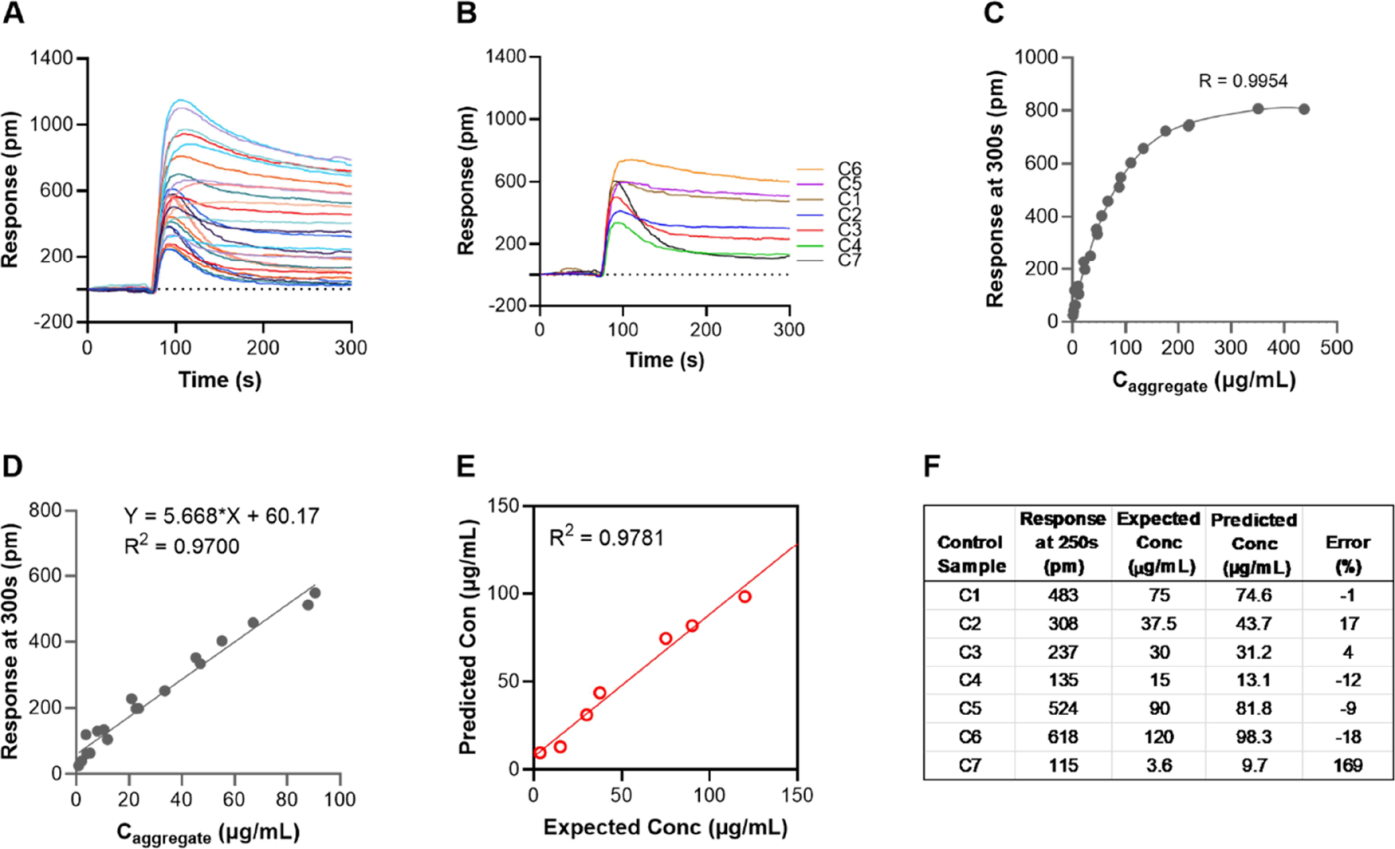
(A) Sensorgrams of 27 samples with varying total IgG concentrations (0.1125, 0.25, and 0.45 mg/mL) and aggregate levels (from 1 up to 98%) used for data fitting. (B) Sensorgrams of seven control samples. All sensorgrams were collected using the same sensor chips. (C) Binding curve obtained from 27 standard samples by plotting responses at 300 s against aggregate concentrations and nonlinear regression fitting. (D) Linear range extracted from the binding curve in (D) (samples with responses at 300 s higher than 550 pm were excluded). (E) Regression correlation between predicted and expected concentrations of aggregate for seven control samples. (F) Response, predicted, and expected concentrations of aggregates.

To further explore the possibilities to also quantify the concentration of aggregates, sensorgrams from 34 different samples with varying aggregate levels (from 1 to 98% corresponding to 1.25 to 440 μg/mL) and total IgG concentrations from 0.125 to 0.45 mg/mL were collected. Twenty-seven sensorgrams (Figure 4A) were used for data fitting and seven sensorgrams (Figure 4B) were left out and used as control samples to evaluate the prediction of aggregate concentrations. Distinct binding patterns were observed for all the samples having different combinations of total concentrations and aggregate levels. For the sample with the lowest aggregate concentration (1.25 μg/mL), the response at the end of the dissociation phase was close to zero (Figure 4A), suggesting that the monomers were completely dissociated from the sensor surface. In contrast, aggregates bound more tightly to the surface, and therefore, the baseline at the end of the dissociation phase (*t* = 300 s) was higher for samples containing higher aggregate concentrations. The responses toward the end of the dissociation phase were therefore anticipated to be solely related to the concentration of aggregates in the samples. Responses at 300 s for the 27 samples showed an excellent correlation to the concentration of aggregates (*R*^2^ = 0.9954, Figure 4C), irrespective of the total IgG concentrations.

The limit of detection (LOD) and limit of quantification (LOQ) were determined from the residual standard deviation (σ) and the slope (*S*) of the calibration curve obtained in the range of 9 to 21 μg/mL. LOD (3.3σ/*S*) and LOQ (10σ/*S*) were approximately 9 and 30 μg/mL, respectively, with a dynamic range of 9–200 μg/mL, corresponding to aggregates levels of 0.2–4.4% in a sample with 4.5 mg/L IgG. At a total IgG concentration of 10 mg/mL, which is a typical concentration in many process steps, aggregate levels ≤0.09% should thus be possible to detect. Methods to further increase the dynamic range to include higher aggregate concentrations could include shorter contact times, lower Protein A surface concentrations, or alternative IgG-binding ligands.

A linear fit (Figure 4D) was used to predict the aggregate concentration of seven control samples with known concentrations. A high correlation (*R*^2^ = 0.9781, Figure 4E) was obtained between predicted and expected (known) concentrations using linear regression analysis with relatively low errors except for sample C7 which had very a high prediction error (169%) as a result of a very low aggregate concentration (3.6 μg/mL) (Figure 4F). An even more extensive data set comprising 65 sensorgrams (Figure S5A) was also collected to further validate the approach for simultaneous quantification of aggregates and monomers, focusing on the lower range of aggregate levels (from 1 to 20%). In addition to generate data for the neural net training, the large number of sensorgrams further confirms the high reproducibility and robustness of the technique.

A simple logarithmic nonlinear fitting using maximum responses and linear fitting using responses at 300 s (Figure S5B,C) was evaluated for the concentration prediction. Good correlation coefficients between predicted and expected concentrations of 11 validated samples were obtained for aggregates (*R*^2^ = 0.8783) and monomers (*R*^2^ = 0.9364) (Figure S5D,E). Mean prediction errors of approximately 11% were obtained for monomers, whereas aggregate predictions gave a mean error of 21% for two samples having concentrations higher than the LOQ (30 μg/mL) (Table S1, Supporting Information).

**Figure 5:**
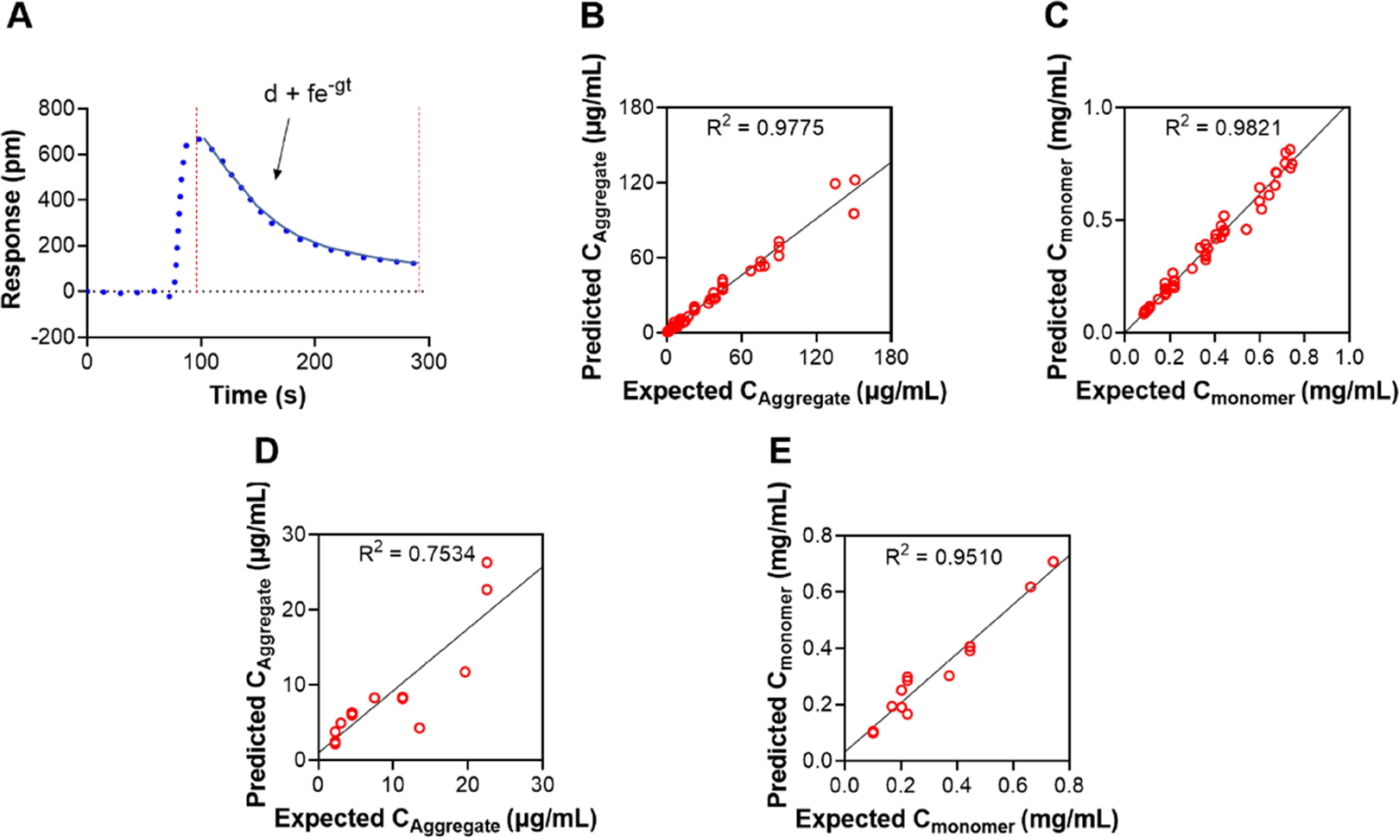
(A) Illustration of the exponential fit using only the dissociation phase. Three fit parameters d, f, and g obtained from the exponential fits were used for building a prediction model using neural network training. (B) and (C) Regression correlations of predicted and expected concentrations of monomer and aggregate for training data set (51 samples). (D) and (E) Regression correlations of predicted and expected concentrations of monomer and aggregate for validation data set (14 samples).

To further improve the accuracy and precision for low aggregate concentrations, a neural network curve fitting approach was explored. The method was first evaluated using only the dissociation phase (Figure 5A). High regression correlation coefficients of expected and predicted concentrations were obtained for both aggregates (*R*^2^ = 0.9775) and monomers (*R*^2^ = 0.9821) for 51 samples in the training set (Figure 5B,C). Cross-validation using the training set gave mean errors of 19 and 6% for aggregate and monomer concentrations, respectively. For the validation set containing 14 samples, aggregate prediction also gave a high correlation (*R*^2^ = 0.7534) between predicted and expected values, even when all the concentrations were lower than 30 μg/mL (Figure 5D). As expected, prediction of monomers was more accurate (*R*^2^ = 0.951) than aggregates due to the substantially higher concentrations of monomers in the samples (Figure 5E). Mean errors calculated from Tables S2 and S3 (Supporting Information) for aggregate and monomer prediction in 14 samples in the validation data set using the dissociation phase were 32 and 14%, respectively. Data fitting and net training using both the association and dissociation phase (Figures S6–S8, Supporting Information) were found to have better monomer prediction with a mean error of 6% but generated a slightly higher mean error (34%) for aggregate prediction compared to net training using only the dissociation phase (Tables S2 and S3, Supporting Information). We expect that the performance of the data fitting can be further improved by increasing the size of the training data set.

## 4 Conclusions

We have demonstrated a fiber optical nanoplasmonic biosensor exploiting avidity effects for rapid and sensitive and simultaneous quantification of monomers and aggregates in the production of therapeutic mAbs. The avidity effects were found to drastically influence the association and dissociation kinetics for binding of the IgG species in the samples to Protein A sensor chips. The effect on the binding was highly dependent on the relative concentrations of aggregates and monomers, which enabled detection of aggregates with an LOD of 9 μg/mL and a LOQ of 30 μg/mL by using only one response data point at 300 s for the analysis. Curve fitting and neural net training using either the dissociation phase or the whole sensorgram significantly improved the accuracy in the detection of concentrations lower than 30 μg/mL. Simultaneous measurements of monomer and aggregate concentrations could also be achieved with high accuracy and precision, and therefore, relative amounts of aggregates can be deduced without the need to measure total mAb concentrations using other methods. The presented approach is both robust and very rapid compared to conventional techniques for aggregate detection. Therefore, the proposed methodology can be used as a rapid tool for monitoring batch variations, product storage stability, or for at-line up- and downstream process monitoring. By further combining the detection concept based on avidity effects and the possibility for in-line integration of the LSPR-sensor technology, this approach can facilitate the development of PAT for real-time detection of IgG monomers and aggregates in downstream bioprocess unit operations.

## Supporting Information Available

Neural network training; Langmuir model; SEC chromatograms; comparison of sensorgrams form mouse IgG2a and human IgG2; principal component analysis; evaluation of monomer and aggregate quantification; data fitting and net training using both the association and dissociation phase; curve-fitting for the dissociation and association phases; and monomer and aggregate prediction (PDF)
