# Supplementary material for: Nanoplasmonic Avidity-Based Detection and Quantification of IgG Aggregates
Source: Anal Chem. 2022 Nov 1;94(45):15754–62. doi: 10.1021/acs.analchem.2c03446 (PMC9670026; doi:10.1021/acs.analchem.2c03446)
Supplement: Supplementary file 1 — ac2c03446_si_001.pdf [file ac2c03446_si_001.pdf]

Supporting Information

Nanoplasmonic Avidity-Based Detection and Quantification of IgG Aggregates

Thuy Tran[1], Erik Martinsson[2], Sergio Vargas[3], Ingemar Lundström[4], Carl-Fredrik Mandenius[5], Daniel Aili[1,]*

[1]Laboratory of Molecular Materials, Division of Biophysics and Bioengineering, Department of Physics, Chemistry and Biology, Linköping University, 581 83 Linköping, Sweden
[2]ArgusEye AB, Spannmålsgatan 55, 583 36 Linköping, Sweden.
[3]Wolfram MathCore AB, Teknikringen 1E, 583 30 Linköping, Sweden.
[4] Sensor and Actuator Systems, Department of Physics, Chemistry and Biology, Linköping University, 581 83 Linköping, Sweden.
[5] Biotechnology, Division of Biophysics and Bioengineering, Department of Physics, Chemistry and Biology, Linköping University, 581 83 Linköping, Sweden.

*Author to whom correspondence should be addressed: daniel.aili@liu.se

Contents

1. Experimental Section 2
Neural Network Training 2
2. Results and Discussions 3
Langmuir Model 3
3. Figures 4
Figure S1. 4
Figure S2. 5
Figure S3. 5
Figure S4. 6
Figure S5. 7
Figure S6. 8
Figure S7. 9
Figure S8. 9
4. Tables 10
Table S1 10
Table S2 10
Table S3 11

1. Experimental Section

Neural Network Training

The 3 fit parameters from the exponential fit of the dissociation curve were used as input for the used neural networks, which, as previously discussed in the experimental section, were used for the prediction of aggregate and monomer concentrations. The neural network was created by joining 7 layers, starting with a linear layer that takes the 3-vectors and ending with a linear layer that outputs 2-vectors, corresponding to the 2 concentrations of interest. The 7 layers alternated between linear and SELU type, with the output of the linear layers being 10-vectors, except for the final 2-vector layer. These sizes and types are all that is needed to generate the neural network object. For this purpose, the function NetChain was used, which simply lists the chosen layers with the discussed types and in the desired order. All that remains before the training itself is initializing the learnable parameters in the layers of the neural network. To do this, we used the function NetInitialize, where we used the random seed 1234 and specified "Kaiming" as the initialization method. This method chooses weights to preserve variance of arrays when propagated through layers. Training was then performed with NetTrain, with a fraction of the data set apart for validation, and where a training time goal of 10 minutes was specified. We also used the fit parameters for both the association and dissociation curves as input for the neural network. These are then 6 fit parameters in total. In that case the initial linear layer of the neural network had 6-vectors as input, while all the remaining specifications were kept the same.

The following lines of Wolfram Language code summarize the discussed details for the training of the neural network:

```
inputSize = 3;
elem = "ScaledExponentialLinearUnit" ;
size = 10;
net = NetChain[{
   LinearLayer[size, "Input" -> inputSize],
   ElementwiseLayer[elem],
   LinearLayer[size, "Input" -> size],
   ElementwiseLayer[elem],
   LinearLayer[size, "Input" -> size],
   ElementwiseLayer[elem],
   LinearLayer[2, "Input" -> size]
   }];
initializedNet = NetInitialize[net, RandomSeeding -> 1234, Method -> "Kaiming"];
trainNet = NetTrain[initializedNet, trainData, All, ValidationSet -> validationData, TimeGoal -> 10*60];
finalNet = trainNet["TrainedNet"];
```

The variable inputSize was changed from 3 to 6 when association and dissociation curves were used simultaneously. The trainData and validationData were assembled in each case storing the fit parameters as inputs and the concentrations as outputs, and later splitting them into training and validation. This split was done in such a way that sufficient variability in the concentrations was included in each set.

2. Results and Discussions

Langmuir Model

The interaction between IgG and protein A can be described by the following reaction and rate equations, based on the Langmuir 1:1 model:

$$IgG + L \leftrightharpoons IgG{:}L \qquad (1)$$

$$\frac{d[IgG{:}L]}{dt} = k_{on} \times [IgG] \times [L] - k_{off} \times [IgG{:}L] \qquad (2)$$

where IgG is the analyte and L represents the IgG-binding ligand (Protein A) on the sensor surface, and k_{on} and k_{off} are the association and dissociation rates, respectively. The observed LSPR responses correlates directly to the surface concentration of the complex [*IgG:L*] and the concentration of unbound IgG ([*IgG*]), which can be assumed to be equal to the IgG concentration in the sample. The free ligand concentration [*L*] correspond to R_{max} - *R*, in which R_{max} is defined as the maximum response obtained when all ligand binding sites are occupied. Equation (2) can be converted to the following differential and integrated rate equations:

$$\frac{dR}{dt} = k_{on} \times C \times (R_{max} - R) - k_{off} \times R \qquad (3)$$

$$R = R_{eq} \times \left(1 - e^{-(k_{on} \times C + k_{off})(t - t_0)}\right) \qquad (4)$$

R_{eq} is the response when the binding between ligand and analyte reaches equilibrium. By setting $k_{on} \times C + k_{off} = k_{obs}$, equation (4) can be finally transformed to

$$\ln \frac{(R_{eq} - R_t)}{R_{eq}} = -k_{obs} \times (t - t_0) \qquad (5)$$

Similarly, the dissociation phase after the formation of the complex can also described by a single-exponential decay of the signal:

$$R_t = R_{eq} \times e^{-k_{off}(t - t_0)} \qquad (6)$$

3. Figures

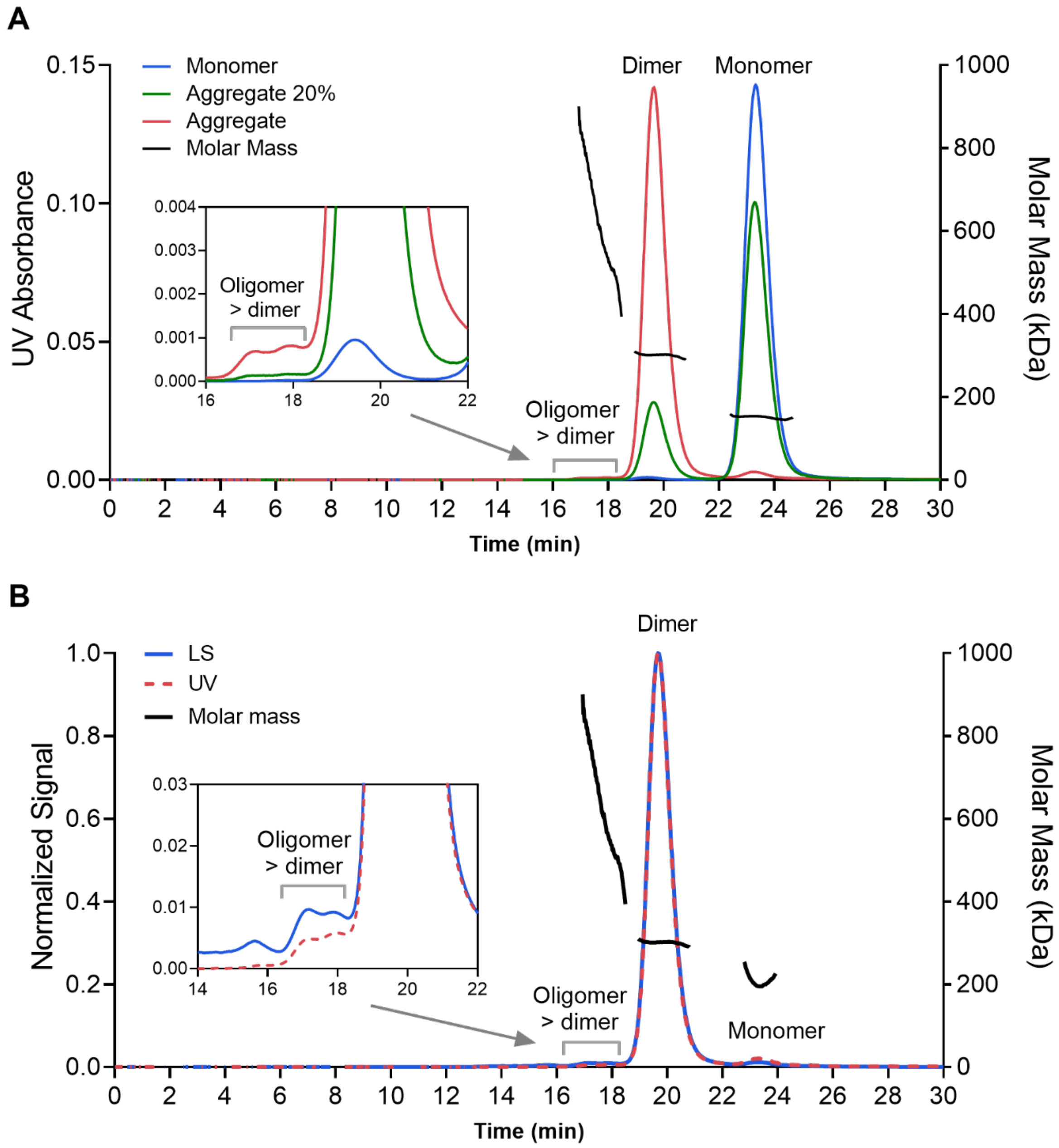

Figure S1. (A) SEC chromatograms of samples with monomers, aggregates and a mixture containing 20% aggregates. Molar mass identification was based on light scattering signals from MALs detection. Total IgG concentration in each sample was 0.45 mg/mL. (B) Normalized signals from light scattering (LS) and UV detection of aggregate sample and molar masses.

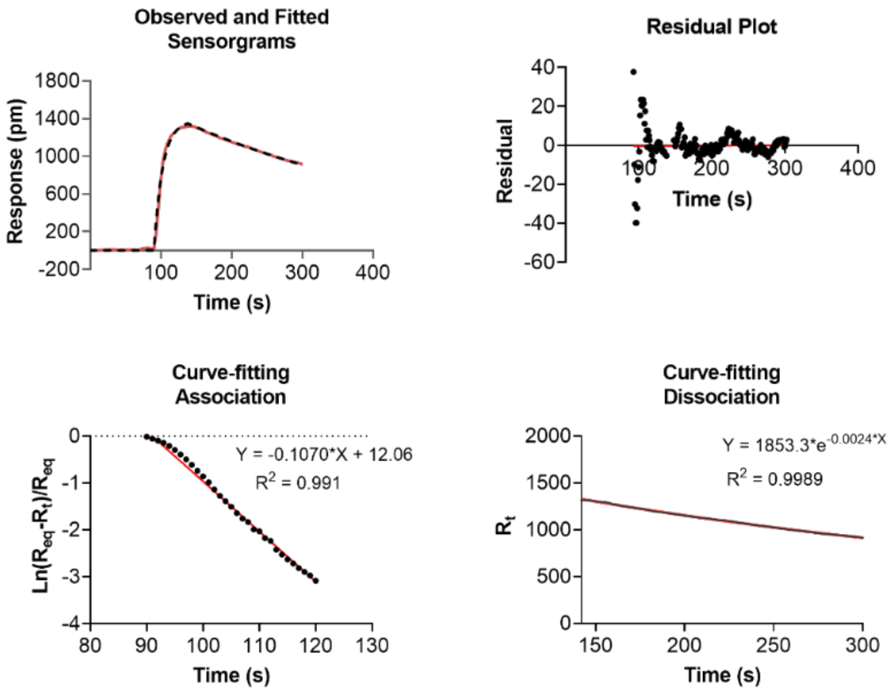

Figure S2. Data fitting using equation (5) and (6) including observed and fitted sensorgrams, residual plot and regression equations with R^2 values for association and dissociation phases using Langmuir 1:1 binding model for aggregate sample (3 μM) at pH 3.8

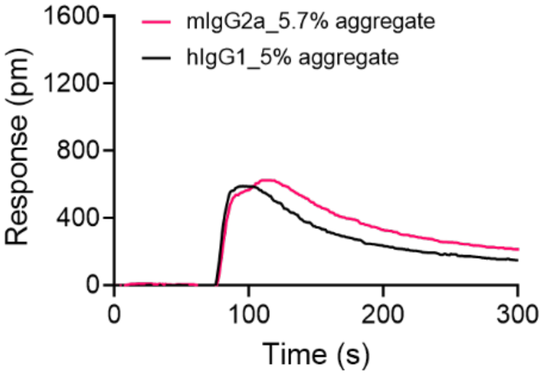

Figure S3. Comparison of binding curves of two samples, a mouse IgG2a (mIgG2a) sample containing 5.7% of aggregates and a human IgG (hIgG1) having 5% of aggregates. Citrate 50 mM, 150 mM NaCl, pH 3.8 was used as the running buffer and total IgG concentrations were 0.45 mg/mL for both samples.

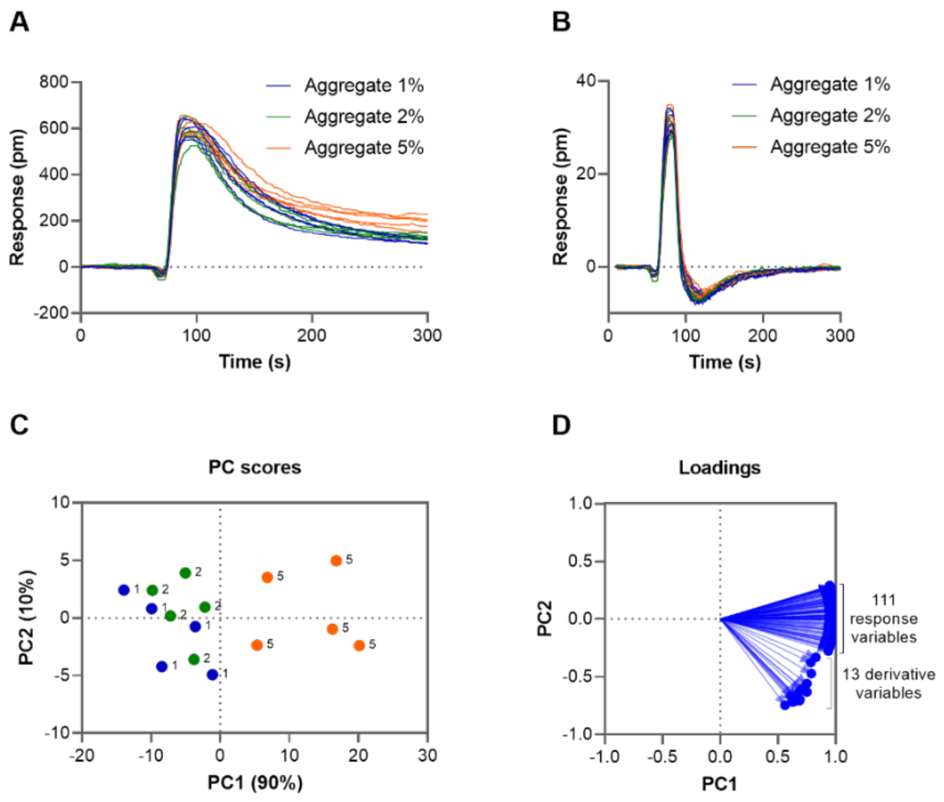

Figure S4. (A) Sensorgrams and (B) first derivatives of three different IgG samples containing 1, 2 and 5% IgG aggregates at pH 3.8. Five replicates from different sensor chips were collected for each sample. (C) Score plot and (D) loading plot from Principal Component Analysis (PCA) using GraphPad Prism software. Responses of 111 data points in the dissociation phase from 190 s to 300 s and 13 derivative points from 110 s to 122 s were used for the analysis.

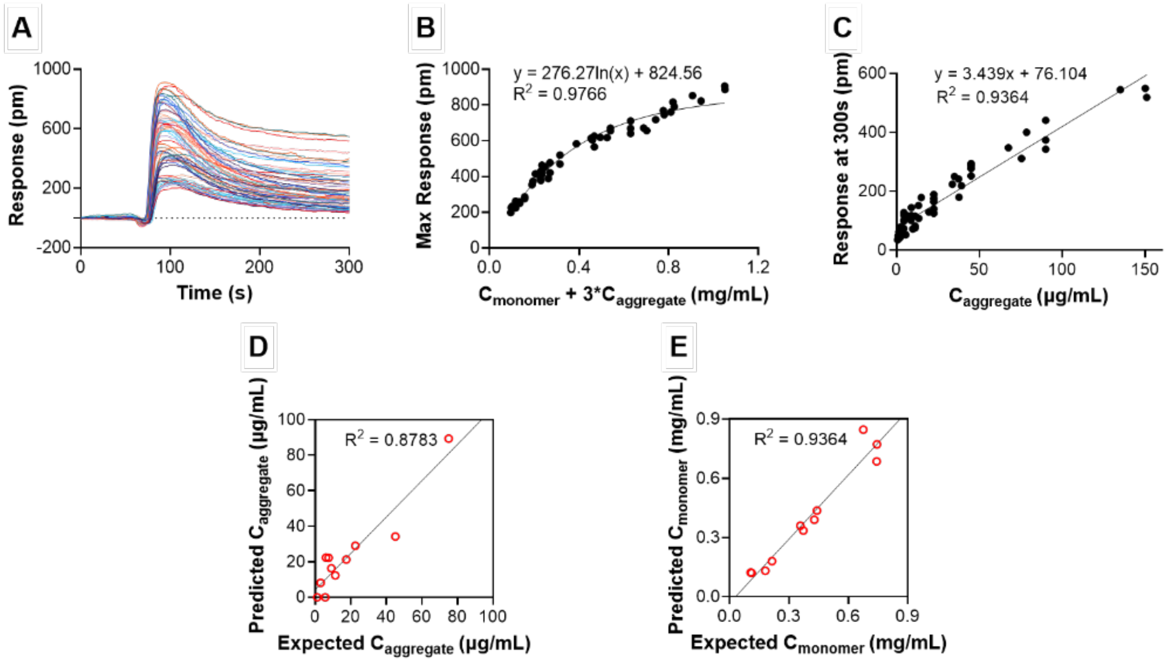

Figure S5. Evaluation of monomer and aggregate quantification using mixtures of purified IgG monomer and aggregate as standards and control samples at pH 3.8. (A) Sensorgrams of 65 samples of varying aggregate (1 - 20%) and total IgG concentrations (0.1125 to 0.75 mg/mL). (B), (C) Non-linear and linear fitting for monomer and aggregate quantification using maximum responses and responses at 300 s. (D), (E) Regression correlations of predicted and expected concentrations of monomer and aggregate for 11 control samples using the fitting described in B and C, respectively.

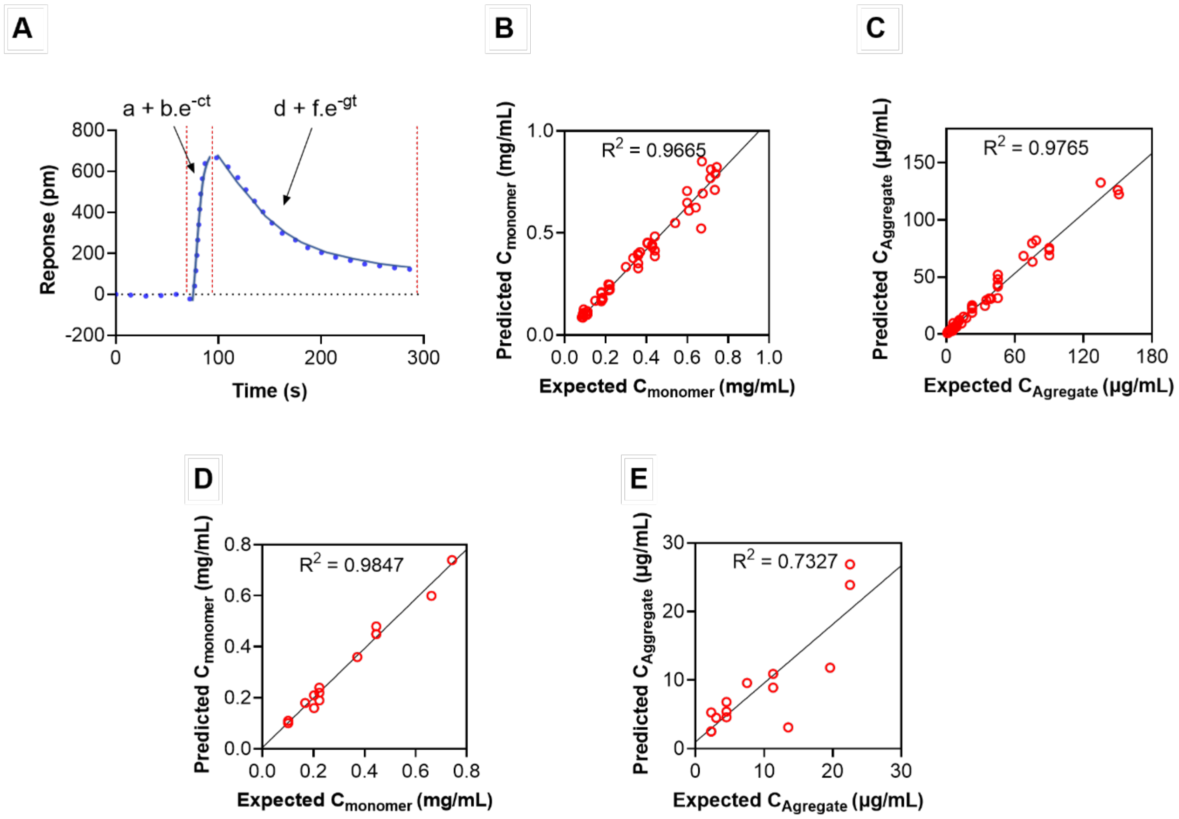

Figure S6. (A) Illustration of the exponential fit using the whole curve including association and dissociation phases. Six fit parameters a, b, c, d, f and g obtained from the exponential fits were used for building a prediction model using neural network training. (B) and (C) Regression correlations of predicted and expected concentrations of monomers and aggregates for training data set (51 samples). (D) and (E) Regression correlations of predicted and expected concentrations of monomers and aggregates for validation data set (14 samples).

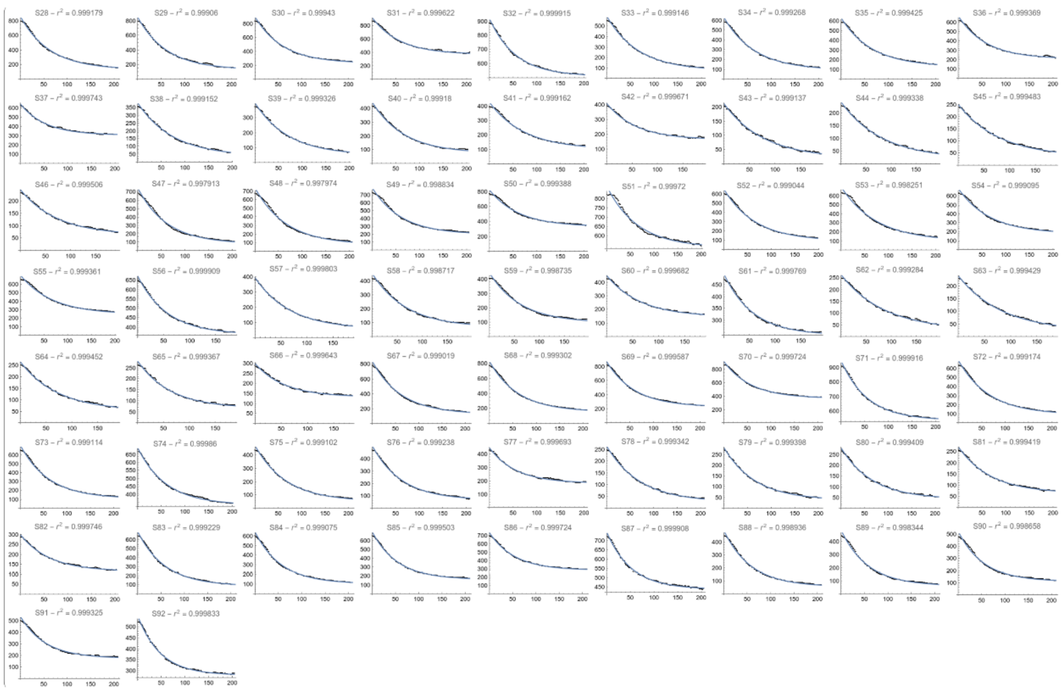

Figure S7. Curve-fitting for the dissociation phases of 65 samples used for neural network training and validation of prediction models.

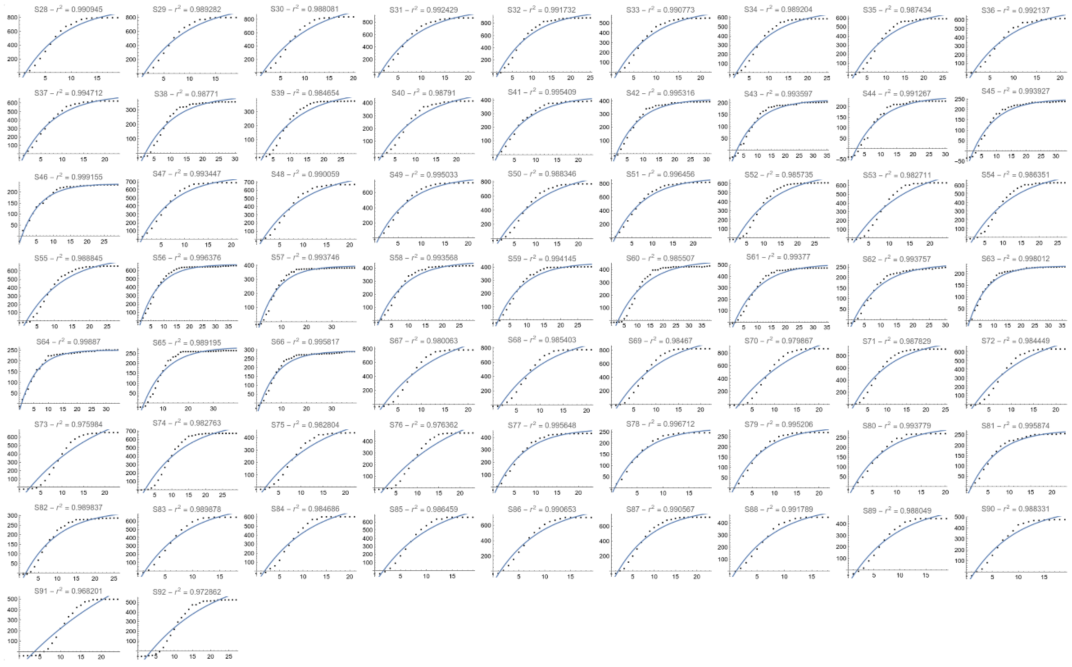

Figure S8. Curve-fitting for the association phases of 65 samples used for neural network training and validation of prediction models.

4. Tables

Table S1. Monomer and aggregate prediction using maximum responses and responses at 300 s.

Sample Name	Expected $C_{aggregate}$ (μg/mL)	Predicted $C_{aggregate}$ (μg/mL)	Expected $C_{monomer}$ (mg/mL)	Predicted $C_{monomer}$ (mg/mL)	Aggregate Prediction Error (%)	Monomer Prediction Error (%)
S37	6.0	22.4	0.74	0.77	273	4
S33	3.0	8.2	0.37	0.33	174	-10
S77	45.0	34.3	0.18	0.13	-24	-27
S73	9.0	16.4	0.44	0.44	82	-1
S59	11.3	12.4	0.21	0.18	10	-16
S70	75.0	89.3	0.68	0.85	19	25
S35	17.5	21.3	0.36	0.36	22	0.7
S85	22.5	29.0	0.43	0.39	29	-9
S63	5.6	0.0	0.11	0.12	-100	14
S78	1.1	0.0	0.11	0.12	-100	8
S67	7.5	22.3	0.74	0.68	197	-8

Table S2. Aggregate prediction of validation data set (14 samples) using neural network training.

		**Net using dissociation phase only**		**Net using all curve**	
Sample	Expected (μg/mL)	Predicted (μg/mL)	Error (%)	Predicted(μg/mL)	Error (%)
S33	3.0	4.9	65	4.5	50
S41	19.6	11.7	-40	11.8	-40
S48	13.5	4.3	-68	3.1	-77
S52	4.5	6.3	39	6.8	50
S57	2.3	3.8	68	5.3	138
S60	22.5	22.7	1	23.9	6
S65	11.3	8.4	-25	10.9	-4
S67	7.5	8.3	11	9.6	28
S72	4.5	6.3	40	5.4	20
S75	2.3	2.2	-2	2.5	12
S81	11.3	8.2	-27	8.9	-21
S83	4.5	6.0	33	4.6	3
S88	2.3	2.5	11	2.5	12
S91	22.5	26.3	17	26.9	20

Table S3. Monomer prediction of validation data set (14 samples) using neural network training.

		**Net using dissociation phase [illegible]**		**Net using all curve**	
Sample	Expected (mg/mL)	Predicted (mg/mL)	Error (%)	Predicted(mg/mL)	Error (%)
S33	0.37	0.30	-19	0.36	-2
S41	0.17	0.19	15	0.18	8
S48	0.66	0.62	-6	0.60	-10
S52	0.45	0.39	-12	0.45	0
S57	0.22	0.17	-25	0.19	-14
S60	0.20	0.19	-6	0.21	3
S65	0.10	0.11	4	0.11	11
S67	0.74	0.71	-5	0.74	0
S72	0.45	0.41	-9	0.45	1
S75	0.22	0.29	28	0.22	-1
S81	0.10	0.10	-2	0.10	-4
S83	0.45	0.41	-9	0.48	7
S88	0.22	0.24	7	0.30	34
S91	0.20	0.16	-22	0.25	24
